# Draft Genome Sequences of Thiorhodococcus mannitoliphagus and Thiorhodococcus minor, Purple Sulfur Photosynthetic Bacteria in the Gammaproteobacterial Family *Chromatiaceae*

**DOI:** 10.1128/MRA.00193-20

**Published:** 2020-04-02

**Authors:** Fabiola A. Aviles, Terry E. Meyer, John A. Kyndt

**Affiliations:** a College of Science and Technology, Bellevue University, Bellevue, Nebraska, USA; b Department of Chemistry and Biochemistry, the University of Arizona, Tucson, Arizona, USA; University of Southern California

## Abstract

We have determined the draft genome sequences of Thiorhodococcus mannitoliphagus and Thiorhodococcus minor for comparison with those of T. drewsii and Imhoffiella purpurea. According to average nucleotide identity (ANI) and whole-genome phylogenetic comparisons, these two species are clearly distinct from the *Imhoffiella* species and *T. drewsii*.

## ANNOUNCEMENT

The purple photosynthetic bacterial family *Chromatiaceae* is fairly large, with more than 72 named species, and the genus *Thiorhodococcus* alone contains about 10 species. The first *Thiorhodococcus* species was described by Guyoneaud et al. (1) as T. minus, which was later changed to T. minor (2). *Thiorhodococcus* species, including *T. drewsii* (3), were reported to be most closely related to the species of *Allochromatium* and *Thiocystis* (4). Thiorhodococcus bheemlicus was split off as Imhoffiella bheemlica, which along with Imhofiella purpurea AK35 are the only species in the genus (5). The genome sequences currently reported are those of *T. drewsii* (6) and *I. purpurea* AK35 (7). We have now produced draft genome sequences for *T. minor* and T. mannitoliphagus (8) to further define the genus and to clarify the position of the *Imhoffiella* species.

Cells of Thiorhodococcus minor DSM 11518 and *T. mannitoliphagus* DSM 18266 were grown and genomic DNA was prepared by the DSMZ culture collection. DNA analysis using Qubit and NanoDrop showed an *A*_260/280_ ratio of 2.1 for *T. mannitoliphagus* and 1.74 for *T. minor*. The sequencing libraries were prepared using the Illumina Nextera DNA Flex library prep kit. The genomes were sequenced with an Illumina MiniSeq using 500 μl of a 1.8 pM library. Paired-end (2 × 150-bp) sequencing generated 2,534,816 reads and 198.3 Mbp for *T. minor* (32× coverage) and 2,624,596 reads and 207.5 Mbp for *T. mannitoliphagus* (33× coverage). Quality control of the reads was performed using FastQC within BaseSpace version 1.0.0 (Illumina), using a k-mer size of 5 and contamination filtering. We assembled the genome *de novo* using SPAdes version 3.10.0 (9) through PATRIC (10). This assembly yielded 389 contigs (>300 bp) and an *N*_50_ value of 60,973 bp for *T. minor*, while *T. mannitoliphagus* was assembled into 525 contigs with an *N*_50_ value of 38,298 bp. The *T. minor* genome had a GC content of 64.8% and was 6,204,734 bp long, and the *T. mannitoliphagus* genome had 62.5% GC content and was 6,310,033 bp long. The genomes were annotated using the RAST tool kit (RASTtk; 11) within PATRIC (10). This showed *T. minor* to have 6,147 coding sequences (CDSs) and 50 tRNAs, and *T. mannitoliphagus* contained 6,471 CDSs and 48 tRNAs. The genomes, when annotated using the NCBI Prokaryotic Genome Annotation Pipeline (PGAP), resulted in 5,426 CDSs for *T. minor* and 5,468 CDSs for *T. mannitoliphagus*. Default parameters were used for all software applications unless otherwise noted.

**FIG 1:**
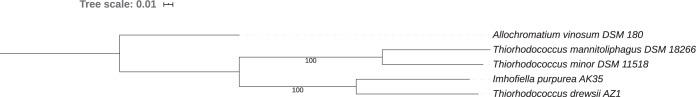
Whole-genome-based phylogenetic tree of all sequenced *Thiorhodococcus* and *Imhoffiella* species. The phylogenetic tree was generated using the codon tree method within PATRIC (10), which used cross-genus families (PGFams) as homology groups. Among these selected genomes, 500 PGFams were found using the CodonTree analysis, and the aligned proteins and coding DNA from single-copy genes were used for RAxML analysis (13, 14). The support values for the phylogenetic tree were generated using 100 rounds of the “Rapid bootstrapping” option in RaxML (13). Allochromatium vinosum was used as an outgroup. iTOL was used for the tree visualization (15).

A JSpecies comparison (12) of average percent nucleotide identity (ANIb) showed 82.3% identity between *T. minor* and *T. mannitoliphagus*. Both genomes gave an ANI of 75% with *Thiorhodococcus drewsii* DSM15006 and 75 to 76% with Imhoffiella purpurea AK35. The ANI numbers for all these genomes are clearly below the proposed 95% cutoff for genome definition of a species (12). *T. drewsii* and *I. purpurea* are also 82% identical to one another, which does not support the separation of these two as separate genera. Whole-genome-based phylogenetic analysis of all of the *Thiorhodococcus* genomes using RAxML within PATRIC (13, 14) showed two distinct groups, with *T. minor* and *T. mannitoliphagus* as the closest relatives and the group of *T. drewsii* and *I. purpurea* more distantly related, consistent with the ANI analysis (Fig. 1). These whole-genome analyses suggest that while *T. minor* and *T. mannitoliphagus* group together, *T. drewsii* could be included in the genus *Imhoffiella* if further sequence, morphological, and physiological studies warrant it, although our studies suggest that all four should remain in the genus *Thiorhodococcus*, which is clearly differentiated from *Allochromatium*.

### Data availability.

These whole-genome shotgun projects have been deposited at DDBJ/ENA/GenBank under the accession numbers JAAIJQ000000000 for Thiorhodococcus minor and JAAIJR000000000 for Thiorhodococcus mannitoliphagus. The versions described in this paper are versions JAAIJQ010000000 and JAAIJR010000000. The raw sequencing reads have been submitted to SRA, and the corresponding accession numbers are SRR11068433 for Thiorhodococcus minor and SRR11068465 for Thiorhodococcus mannitoliphagus.
